# Supplementary material for: Venomix: a simple bioinformatic pipeline for identifying and characterizing toxin gene candidates from transcriptomic data
Source: PeerJ. 2018 Jul 31;6:e5361. doi: 10.7717/peerj.5361 (PMC6074769; doi:10.7717/peerj.5361)
Supplement: Supplemental Information 3 [file peerj-06-5361-s003.gz › FinalOutput_E-20/Kunitz-type_serine_protease_inhibitor_Kunitz-1_1/finaltree.pdf]

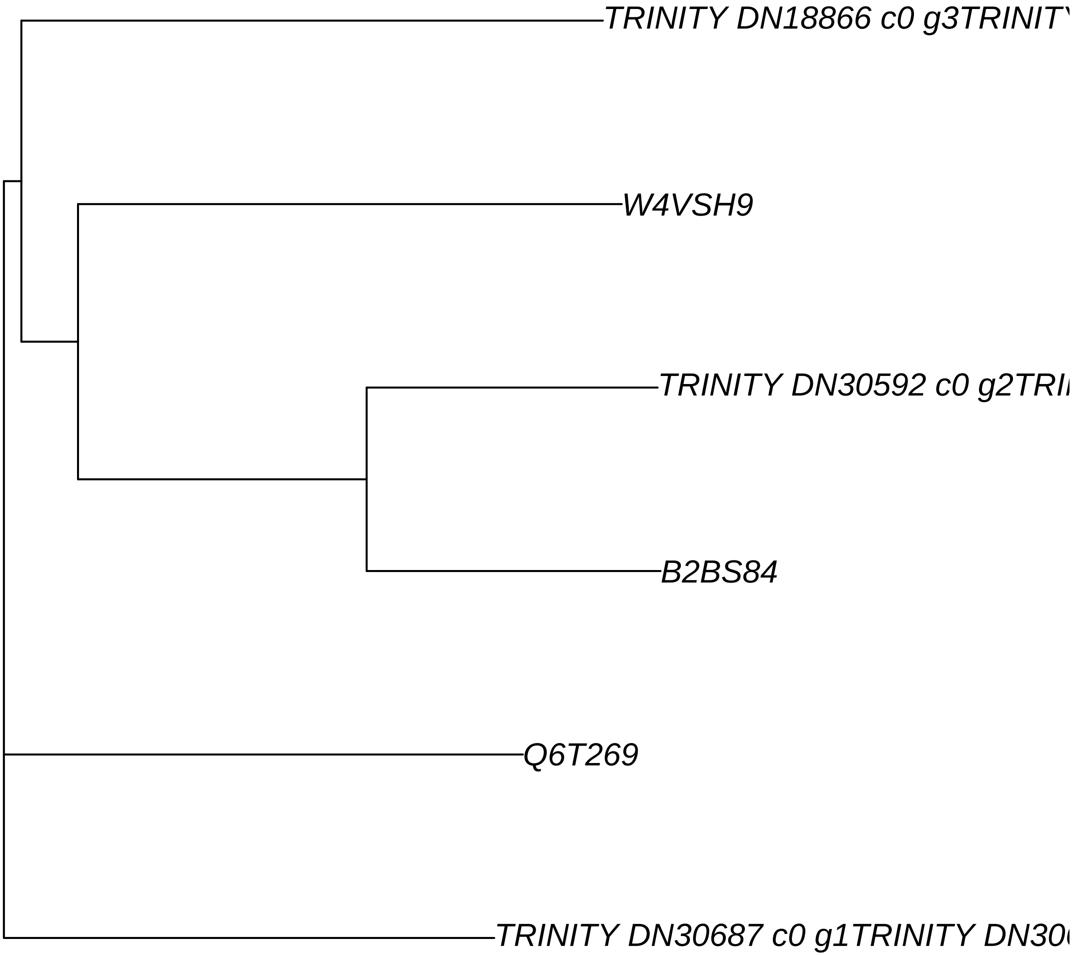
TRINITY DN18866 c0 g3TRINITY
W4VSH9
TRINITY DN30592 c0 g2TRI
B2BS84
Q6T269
TRINITY DN30687 c0 g1TRINITY DN30
